# Nonfouling Coatings from Synthetic Intrinsically Disordered Proteins

**DOI:** 10.1002/smll.202504365

**Published:** 2025-06-30

**Authors:** Chuanbao Zheng, Yulia Shmidov, Anastasia K. Varanko, Sonal Deshpande, Yunqi Yang, Daniel M. Shapiro, Zhisen Zhang, Han Zuilhof, Ashutosh Chilkoti, Renko de Vries

**Affiliations:** ^1^ Physical Chemistry and Soft Matter Wageningen University & Research Wageningen 6708WE The Netherlands; ^2^ Laboratory of Organic Chemistry Wageningen University & Research Wageningen 6708WE The Netherlands; ^3^ Department of Biomedical Engineering Duke University Durham NC 27708 USA; ^4^ Research Institute for Biomimetics and Soft Matter Fujian Provincial Key Laboratory for Soft Functional Materials Research Department of Physics Xiamen University Xiamen 361005 China; ^5^ College of Biological and Chemical Engineering Jiaxing University Jiaxing 314001 China

**Keywords:** antifouling, gold surface, human serum, intrinsically disordered polypeptides, solid‐binding peptides

## Abstract

The antifouling performance of a previously developed triblock protein, ***B‐M‐E*** is optimized, that self‐assembles on gold surfaces to form a nonfouling layer by modifying the sequence of its ***E*** block. In this protein, ***B*** is a gold‐binding domain, ***M*** is a trimerization domain, and ***E*** is a synthetic intrinsically disordered protein (IDP) that confers nonfouling behavior. To identify optimal sequences for the nonfouling ***E*** block, a few candidate IDPs are screened that provide a proxy for nonfouling behavior, such as extending the half‐life of their fusion partners in systemic circulation and sequences that promote soluble expression of their fusion partners in *E coli*. One IDP is identified with the sequence [(GAGAIP)_3_‐(GAGEIP)]_4_ as the ***E*** block in ***B‐M‐E*** brush on gold, forming a nonfouling coating with performance comparable to a self‐assembled monolayer (SAM) of a tetraethylene glycol‐terminated alkanethiol on gold. These ***B‐M‐E*** brushes also render gold surfaces resistant to *E. coli* attachment for at least seven days. The ***B‐M‐E*** protein can be synthesized at scale in bacterial expression systems using the upstream fermentation and downstream purification capabilities of the biotechnology industry. It may provide a useful and robust alternative to existing nonfouling coatings based on small molecules or synthetic polymers.

## Graphical abstract

The antifouling performance of a triblock protein, ***B‐M‐E*** is optimized, by screening intrinsically disordered proteins (IDPs) for its ***E*** block. The best candidate, [(GAGAIP)₃‐(GAGEIP)]₄, forms a nonfouling layer on gold comparable to PEG‐based SAMs and resists *E. coli* attachment for at least seven days. Additionally, ***B‐M‐E*** production is scalable via bacterial expression.

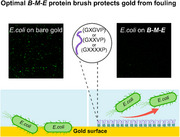

## 1 Introduction

The nonspecific adsorption of proteins and cells on solid surfaces, commonly referred to as fouling, has a significant and often detrimental effect on medical and diagnostic devices, industrial water filtration, and in the food, dairy, and beverage industry.^[^ ^1^, ^2^ ^]^ For example, adsorption of proteins or micro‐organisms on the surface of medical implants can lead to infection and inflammation.^[^ ^3^ ^]^ In molecular diagnostic devices, nonspecific adsorption can reduce the sensitivity and specificity.^[^ ^4^ ^]^

To address fouling with microorganisms, surfaces can be functionalized with biocides,^[^ ^5^, ^6^ ^]^ but this is not desirable as these biocidal molecules are often toxic. Alternatively, covalently attached brushes of hydrophilic synthetic polymers have been found to be effective in preventing the nonspecific adsorption of biomolecules and microorganisms.^[^ ^7^, ^8^, ^9^ ^]^ Hydrophilic synthetic polymer brushes of poly(oligoethylene glycol methacrylate)(POEGMA),^[^ ^10^, ^11^, ^12^ ^]^ zwitterionic polymers,^[^ ^13^, ^14^, ^15^ ^]^ polyethylene glycol^[^ ^16^, ^17^, ^18^, ^19^ ^]^ and other chemistries demonstrated varying degrees of nonfouling behavior on a range of solid materials.^[^ ^20^, ^21^, ^22^, ^23^, ^24^, ^25^ ^]^

We have recently developed a complementary approach to create nonfouling brushes. This approach uses designer proteins that self‐assemble into dense brushes that display synthetic intrinsically disordered proteins (SynIDPs) at the solid‐water interface on silica, gold, and polystyrene surfaces.^[^ ^26^, ^27^, ^28^ ^]^ These designer proteins have a ***B‐M‐E*** domain structure, where ***B*** is a surface‐binding domain,^[^ ^29^ ^]^ ***M*** is a trimerization domain that enhances the strength of surface attachment via multivalency, and ***E*** is a hydrophilic SynIDP that confers nonfouling behavior to the ***B‐M‐E*** brushes. In previous studies,^[^ ^27^ ^]^ we explored elastin‐like polypeptides (ELPs)^[^ ^30^, ^31^, ^32^, ^33^, ^34^ ^]^ with hydrophilic guest residues as the ***E*** block and found that the coating brushes prevent the nonspecific binding of diluted serum proteins on gold surfaces.

Here, we build upon these studies to screen SynIDPs that we hypothesized would have superior nonfouling behavior than ELPs. To identify candidate SynIDPs, we used two proxies for nonfouling behavior based on similar physicochemical requirements: high solvation to enhance interface hydration strength and high conformational freedom to minimize foulant binding sites. The first example is zwitterionic polypeptides (ZIPPs), a class of recombinant polypeptides known to extend the plasma half‐life of their fusion partners.^[^ ^35^, ^36^, ^37^ ^]^ Another example is solubility tags, which promote the soluble heterologous expression and prevent aggregation of their fusion partners. This screening was recently performed for a library of SynIDPs, leading to the identification of new polypeptide sequences with high activity as solubility tags.^[^ ^38^ ^]^ We demonstrate herein that a new SynIDP sequence [(GAGAIP)_3_‐(GAGEIP)]_4_, previously identified as a hyper‐soluble IDP with good activity as a solubility tag,^[^ ^38^ ^]^ also exhibits excellent antifouling activity when used as the antifouling ***E*** block in a ***B‐M‐E*** fusion protein. When ***B‐M‐E*** protein is assembled as a polypeptide brush on a gold surface, it provides nonfouling activity comparable to an oligoethylene glycol‐terminated self‐assembled monolayer (SAM) on gold, which is considered one of the most protein‐resistant surface coatings on gold.^[^ ^10^, ^39^ ^]^

## 2 Results and Discussion

### 2.1 Design, Synthesis, and Characterization of *B‐M‐E* Proteins

**Figure 1:**
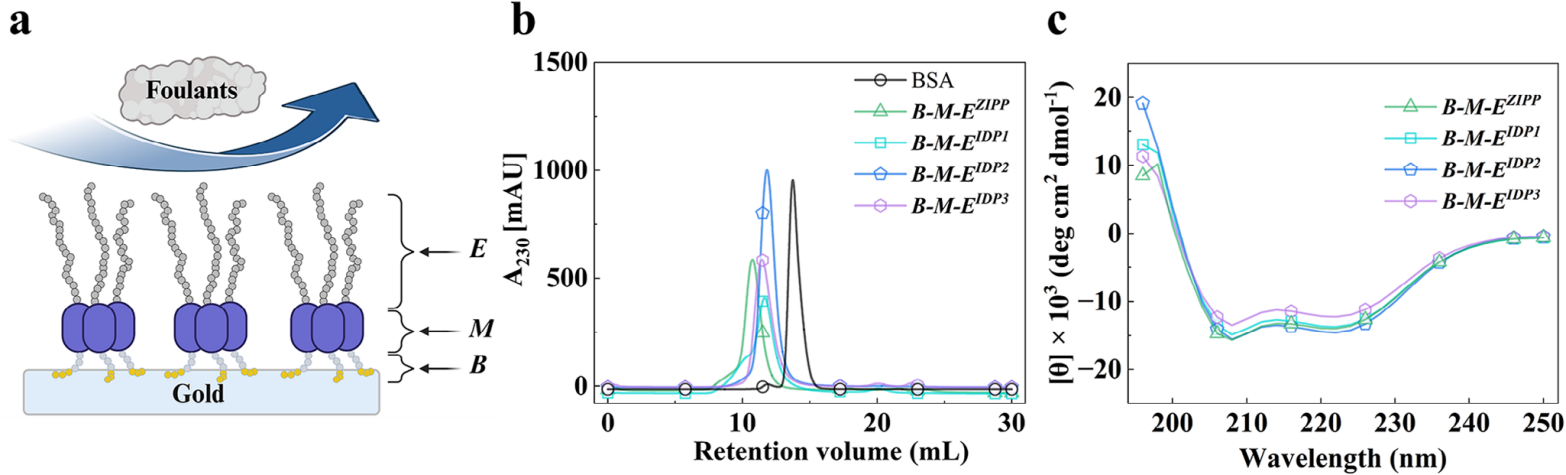
a) Schematic illustration of the self‐assembly of ***B‐M‐E*** proteins into polypeptide brushes on a gold surface. b) Analytical SEC of ***B‐M‐E*** *^ZIPP^*, ***B‐M‐E*** *^IDP1^*, ***B‐M‐E*** *^IDP2^*, ***B‐M‐E*** *^IDP3^* and BSA as a reference. Absorbance at 230 nm as a function of retention volume. The molecular weight of the ***B‐M‐E*** proteins is ≈43 kDa. The molecular weight of BSA is ≈66 kDa. c) CD spectra of the ***B‐M‐E*** proteins at 10 µM in PBS, at 20 °C. Green line with triangles, cyan line with squares, blue line with pentagons, and purple line with hexagons are ***B‐M‐E*** *^ZIPP^*, ***B‐M‐E*** *^IDP1^*, ***B‐M‐E*** *^IDP2^*, and ***B‐M‐E*** *^IDP3^*, respectively.

**Figure** **1**a shows a schematic representation of ***B‐M‐E*** protein molecules assembled into a polypeptide brush on a gold surface. The ***B*** domain consists of a His‐tag (H_6_SSG) located at the N‐terminus, a gold‐binding sequence MHGKTQATSGTIQS,^[^ ^29^, ^40^ ^]^ and a short ELP linker (GSGVP)_3_. The latter connects to the multimerization domain ***M***. The multimerization domain ***M*** is a computationally designed trimerization domain denoted as *HR*00*C*32 that is purely *α*‐helical and highly thermostable.^[^ ^41^ ^]^ A list with basic features of the ***E*** domain in the ***B‐M‐E*** protein constructs (and a ***B‐M*** control construct) used in this study is shown in **Table** **1**. The DNA and amino acid sequences of all constructs are in Tables S1 and S2 (Supporting Information), respectively. Proteins were recombinantly synthesized in *E. coli* and purified using standard chromatography methods. Protein purity was confirmed by sodium dodecyl‐sulfate polyacrylamide gel electrophoresis (SDS‐PAGE), see Figure S1 (Supporting Information). Matrix‐assisted laser desorption/ionization time of flight (MALDI‐TOF) mass spectrometry was used to confirm that these proteins had the expected molar mass (Figure S2 and Table S3, Supporting Information).

**Table 1:** List of *B‐M‐E* proteins used in this study.

Name	*E* block sequence	*E* block length^a)^
*B‐M*	–	0
*B‐M‐E^Z6^*	(GDGVPGKGVP)_3_	30
*B‐M‐E^Z10^*	(GDGVPGKGVP)_5_	50
*B‐M‐E^ZIPP^*	(VPKEG)_20_	100
*B‐M‐E^IDP1^*	(GQSGLP)_16_	96
*B‐M‐E^IDP2^*	(GTHGTP)_16_	96
*B‐M‐E^IDP3^*	[(GAGAIP)_3_‐(GAGEIP)]_4_	96

^a)^ *E* block length is showing the number of amino acids in the *E* block.

Figure 1b shows analytical size exclusion chromatography (SEC) data for the constructs ***B‐M‐E*** *^ZIPP^*, ***B‐M‐E*** *^IDP^* ^1^, ***B‐M‐E*** *^IDP^* ^2^, and ***B‐M‐E*** *^IDP^* ^3^ described above. Retention volumes are similar to those found earlier for ***B‐M‐E*** *^Z^* ^20[^ ^27^ ^]^ and correspond to molecular weights larger than those expected for monomers, indicating the expected trimerization in solution indeed occurs. Additional evidence for trimerization was provided by native PAGE (Figure S3a, Supporting Information) and the trimeric structure predicted by AlphaFold2 via ColabFold^[^ ^42^ ^]^ for the ***B‐M‐E*** *^IDP^* ^3^ protein (Figure S3b, Supporting Information). Figure 1c illustrates the circular dichroism (CD) spectra for the constructs ***B‐M‐E*** *^ZIPP^*, ***B‐M‐E*** *^IDP^* ^1^, ***B‐M‐E*** *^IDP^* ^2^, and ***B‐M‐E*** *^IDP^* ^3^ in phosphate‐buffered saline (PBS). These spectra are similar to those of ***B‐M‐E*** *^Z^* ^20^ studied previously ^[^ ^27^ ^]^ and confirm a predominantly *α*‐helical secondary structure, suggesting that the ***M*** domain is properly folded in the ***B‐M‐E*** proteins. CD spectra for ***B‐M‐E*** *^Z^* ^10^, ***B‐M‐E*** *^Z^* ^6^, and ***B‐M***, which similarly exhibit a predominantly *α*‐helical secondary structure, are shown in Figure S4 (Supporting Information).

### 2.2 Optimal Length of *E* Block

Previously, we found^[^ ^27^ ^]^ that antifouling performance for ***B‐M‐E*** *^Z^* ^20^ brushes could not be improved with longer ***E*** blocks. Here, we first test whether ***E*** *^Z^* ^20^ is indeed the optimal length, or that maybe shorter ***E*** blocks give better antifouling performance.

We used quartz crystal microbalance with dissipation (QCM‐D) to measure the protein adsorption on brushes of ***B‐M‐E*** *^Z10^*, ***B‐M‐E*** *^Z6^*, and ***B‐M*** on gold surfaces from 10% human serum (HS). The results were compared to those previously obtained for ***B‐M‐E*** *^Z^* ^20^.^[^ ^27^ ^]^ QCM‐D experiments were performed as follows: 1) a gold‐coated QCM‐D sensor was equilibrated with PBS, 2) coating proteins were injected, 3) rinsed with PBS, 4) protein adsorption was initiated by injecting 10% HS, followed by 5) a final rinse with PBS. The fouling percentage was calculated from the frequency changes measured in QCM‐D (Equation 1, in the experimental section). The fouling percentage on the bare gold surface was normalized to 100%, as illustrated in Figure S5a (Supporting Information).

**Figure 2:**
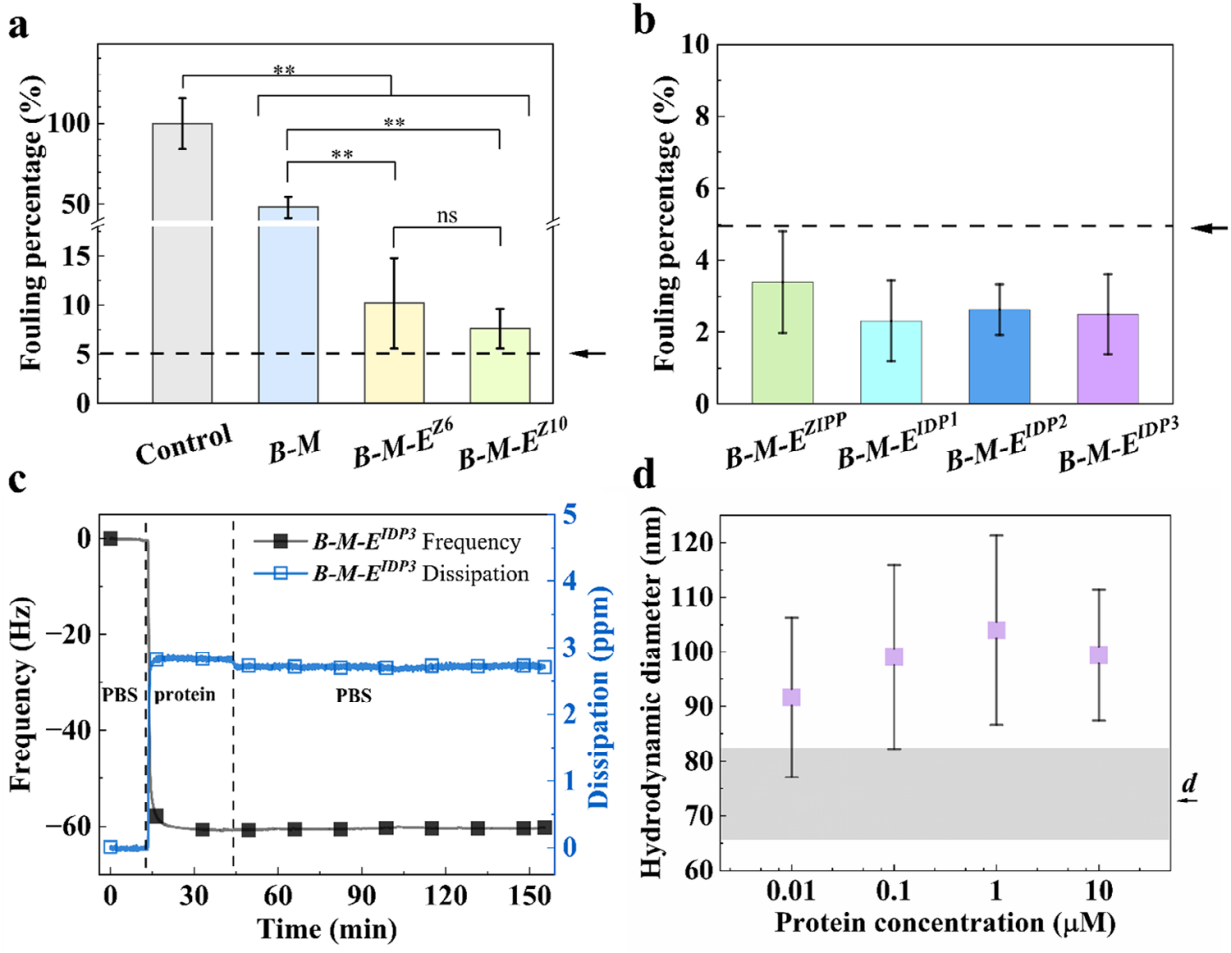
Quantitative analysis of QCM‐D fouling assay of ***B‐M‐E*** coated gold surfaces against 10% HS. a) Fouling percentage of ***B‐M‐E*** proteins. The dashed line is the fouling percentage of ***B‐M‐E*** *^Z^* ^20^.^[^ ^27^ ^]^ b) Influence of ***E*** sequence on nonfouling performance. The difference between samples is not significant. c) QCM‐D frequency and dissipation data of ***B‐M‐E*** *^IDP^* ^3^ coating layer on the gold surface as a function of washing time with PBS. d) Hydrodynamic diameter *D* (nm) of ***B‐M‐E*** *^IDP^* ^3^ coated GNPs as a function of the concentration of ***B‐M‐E*** *^IDP^* ^3^. The *D* of the uncoated GNPs of 73.9 ± 8.3 nm (±SD) is indicated by the gray bar. ***B‐M‐E^IDP3^*** samples were prepared at a concentration of 10 µM and diluted in PBS. Data were analyzed using one‐way ANOVA with post‐hoc Tukey HSD. ^**^ *p <*0.01, ^*^ *p <* 0.05, ns *p >*0.05.

The fouling percentages shown in **Figure** **2**a clearly show that the fouling decreases upon increasing the length of the ***E*** block from 0 to 10 pentamer repeats. However, the fouling level for ***B‐M‐E*** *^Z10^*, ***B‐M‐E*** *^Z6^* remains higher than the previously measured value for ***B‐M‐E*** *^Z^* ^20^ with 20 pentamer repeats.^[^ ^27^ ^]^ The complete set of QCM‐D data for ***B‐M***, ***B‐M‐E*** *^Z^* ^6^, and ***B‐M‐E*** *^Z^* ^10^ is in Figure S5b–d (Supporting Information). These results confirm that a length of 20 pentamers, or 100 amino acids for the ***E*** block, is optimal. Hence, we chose a length of ≈100 amino acids as the ***E*** block for all the new candidate SynIDPs studied herein.

### 2.3 Optimal Sequence of *E* Block

Next, we used QCM‐D to measure the antifouling performance of ***B‐M‐E*** *^ZIPP^*, ***B‐M‐E*** *^IDP^* ^1^, ***B‐M‐E*** *^IDP^* ^2^, and ***B‐M‐E*** *^IDP^* ^3^ with 10% HS as the foulant. Figure 2b shows the fouling percentage for each SynIDP brush and is compared to the value found previously for ***B‐M‐E*** *^Z^* ^20^ (dashed line).^[^ ^27^ ^]^ The new sequences perform better than ***B‐M‐E*** *^Z^* ^20^. However, based on analysis using one‐way ANOVA with post‐hoc Tukey HSD, the differences in the fouling percentage between the four new sequences are not statistically significant with 10% HS as the foulant, probably due to the foulant not being challenging enough (low foulant concentration). To assess the stability of the coating layer, ***B‐M‐E*** *^IDP^* ^3^ was chosen as a representative nonfouling coating with respect to rinsing over time. Figure 2c shows the frequency and dissipation measured by QCM‐D during coating formation and the subsequent PBS rinsing step. There is no change in signal during prolonged rinsing, indicating good stability on the gold surface.

Subsequently, we used dynamic light scattering (DLS) to measure the hydrodynamic diameter (*D*) of monodisperse gold nanoparticles (GNPs) before and after incubation with increasing concentrations of ***B‐M‐E*** *^IDP3^*, to determine the thickness of the polypeptide brush coating layer in PBS. The *D* of the bare GNP is *d* = 73.9 ± 8.3 nm. The *D* of coated particles, as a function of the concentration of ***B‐M‐E*** *^IDP3^*, is shown in Figure 2d, and is similar to ***B‐M‐E*** *^ZIPP^*, ***B‐M‐E*** *^IDP1^*, and ***B‐M‐E*** *^IDP2^* (Figure S6a,b, Supporting Information). For ***B‐M‐E*** *^IDP3^*, the increase in *D* due to the protein coating stabilizes at a concentration of ≈1 µM. Assuming that the effective particle diameter is *D* = *d* + 2*h*, where *h* is the thickness of the polypeptide brush, we arrive at an estimated thickness of the polypeptide brush *h* ≈12–16 nm. Similar values were found for ***B‐M‐E*** *^ZIPP^*, ***B‐M‐E*** *^IDP1^*, and ***B‐M‐E*** *^IDP2^* (Table S4, Supporting Information) and the previously studied ***B‐M‐E*** *^Z20^*.^[^ ^27^ ^]^

### 2.4 Protein Adsorption from 100% HS

**Figure 3:**
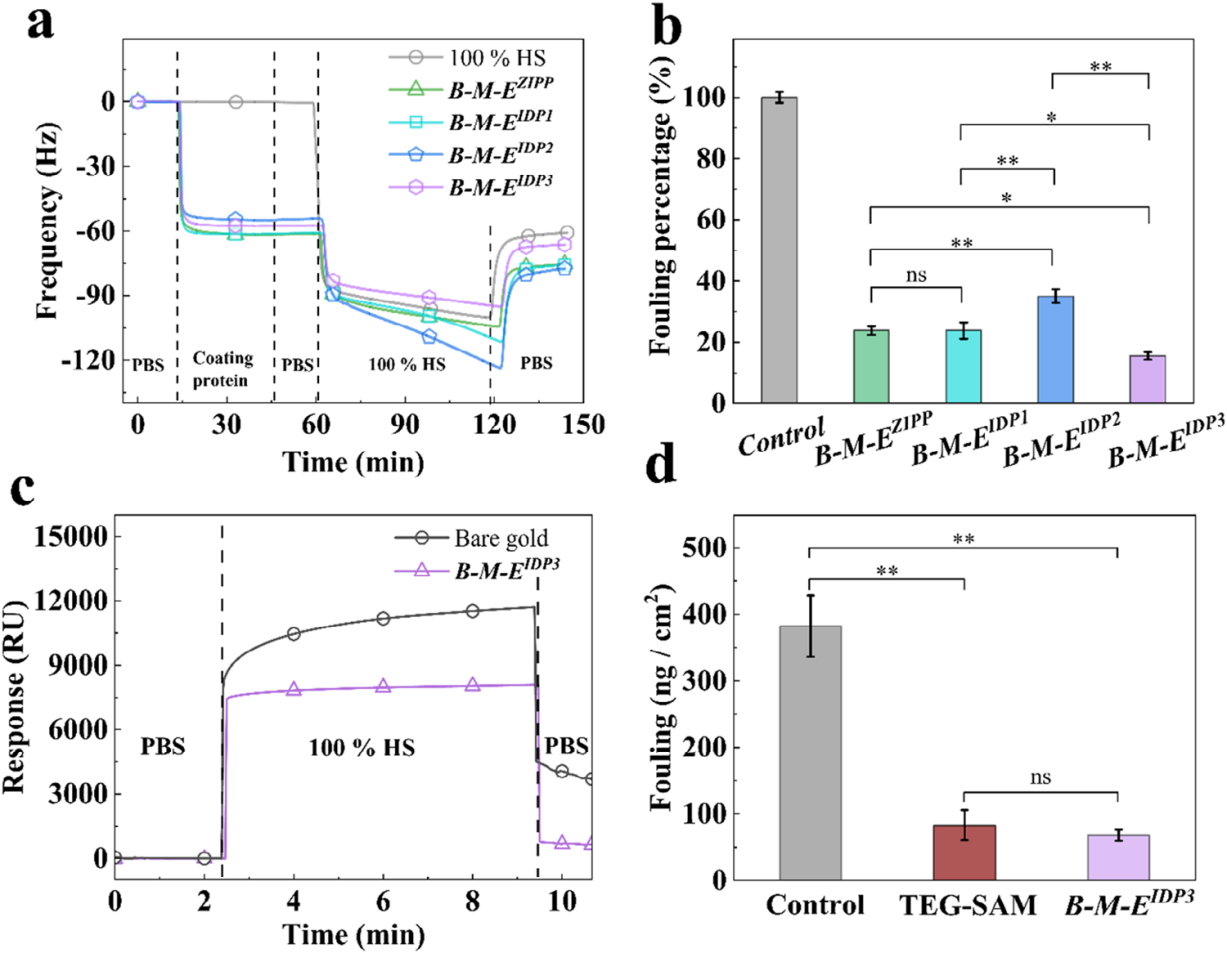
QCM assay of adsorption of undiluted HS on ***B‐M‐E*** coated gold surface. a) QCM‐D frequency data of 100% HS (human serum) adsorption on bare gold (gray line with circles) and gold surface coated with ***B‐M‐E*** *^ZIPP^* (green line with triangles,) ***B‐M‐E*** *^IDP1^* (cyan line with squares), ***B‐M‐E*** *^IDP2^* (blue line with pentagon), and ***B‐M‐E*** *^IDP3^* (purple line with hexagons). b) Fouling percentage of undiluted HS fouling on bare gold surfaces and surfaces coated with different ***B‐M‐E*** proteins calculated from QCM data. (n = 3) ^**^ *p <* 0.01, ^*^ *p <* 0.05, ns *p >*0.05 (one‐way ANOVA with post‐hoc Tukey HSD). c) SPR sensorgrams for the binding of undiluted HS on bare gold (black line with circles) and gold coated with ***B‐M‐E*** *^IDP3^* (purple line with triangles), followed by PBS wash. d) Comparison of fouling on bare gold, bare gold functionalized with TEG‐SAM layer, and ***B‐M‐E*** *^IDP3^*. The data represent the mean ± standard deviation (n = 3) for the SPR data. ^**^ *p <* 0.01, ^*^ *p <* 0.05, ns *p >*0.05 (one‐way ANOVA with post‐hoc Tukey HSD).

Having identified improved sequences for the antifouling block ***E***, we continued to challenge coatings with more aggressive foulants than 10% HS. First, we measured adsorption from undiluted HS (100% HS). **Figure** **3** shows the frequency change in QCM‐D for ***B‐M‐E*** *^ZIPP^*, ***B‐M‐E*** *^IDP1^*, ***B‐M‐E*** *^IDP2^*, and ***B‐M‐E*** *^IDP3^*. The results in Figure 3a demonstrate that the ***B‐M‐E*** proteins rapidly coat the gold surface, with no desorption observed during PBS rinsing. Injection of 100% HS causes significant adsorption on both the bare gold surface and the surfaces coated with ***B‐M‐E*** proteins. A subsequent rinse with PBS removes the weakly bound serum proteins. The remaining, more strongly absorbed serum proteins are considered fouling. Fouling percentages derived from these QCM‐D experiments are shown in Figure 3b. Statistical analysis reveals that the best antifouling was achieved by ***B‐M‐E*** *^IDP^* ^3^ coatings, with a fouling of 15.5 ± 1.2% as compared to the reference bare gold (set as 100%). Additional QCM‐D dissipation data for these experiments are given in Figure S7 (Supporting Information). It is worth noting that ***B‐M‐E*** *^IDP^* ^2^ has significantly worse antifouling performance when compared to the other tested constructs against 100% HS. In addition to histidine's known ability to bind divalent metal ions,^[^ ^43^ ^]^ it has been previously shown that the histidine rich region in histidine rich glycoprotein is responsible for binding various proteins in HS.^[^ ^44^ ^]^ Therefore, we assume that having histidine in the repeating sequence of IDP2 leads to the observed decreased antifouling performance when compared to the other tested constructs.

While QCM‐D is a convenient technique to monitor protein adsorption on solid surfaces, translating the frequency change to the adsorbed mass of protein is straightforward only if the adsorbed layer moves rigidly with the oscillating sensor surface. In that case, the adsorbed mass can be calculated with confidence using the linear Sauerbrey relation.^[^ ^45^ ^]^ However, the highly hydrated polypeptide brushes do not necessarily move in synchrony with the oscillating crystal, making accurate estimation of the adsorbed mass challenging. Alternatively, surface plasmon resonance (SPR) measures the absolute adsorbed amounts ^[^ ^46^ ^]^ but requires gold as a sensing surface.

We therefore turned to SPR to quantitatively compare the protein resistance of ***B‐M‐E*** *^IDP^* ^3^ coatings with a positive control, a commercially available tetraethylene glycol‐terminated alkanethiol SAM (TEG‐SAM) on gold, as it is exceptionally protein‐resistant.^[^ ^47^, ^48^ ^]^ Figure 3c shows representative SPR sensorgrams for bare gold and ***B‐M‐E*** *^IDP^* ^3^ coated gold sensor chips. Similar sensorgrams for TEG‐SAM sensor chips and sensorgrams for multiple consecutive injections of foulants and rinsing steps are in Figure S8 (Supporting Information). In Figure 3d, the absolute value of adsorbed serum proteins on bare gold, TEG‐SAM, and ***B‐M‐E*** *^IDP^* ^3^ coated gold is 380 ± 50 ng cm^−2^, 80 ± 20 ng cm^−2^, and 70 ± 10 ng cm^−2^, respectively. From these data, we conclude that the protein resistance of ***B‐M‐E*** *^IDP^* ^3^ coating is at least comparable to that of commercially available TEG‐terminated SAM on gold. We note however, that the fouling level of ***B‐M‐E*** *^IDP^* ^3^ coating is relatively high compared to the fouling level on oligoethylene glycol‐terminated SAMs reported by Whitesides ^[^ ^49^, ^50^ ^]^ and POEGMA.^[^ ^10^, ^11^, ^12^, ^51^ ^]^ We speculate that the discrepancy with the results reported by Whitesides and coworkers may be related to the method of preparation of the TEG‐SAM. The TEG‐SAM on gold we used in this study was obtained from a commercial supplier, and the self‐assembly conditions and solvent used to prepare the SAM may well be different from those used by Whitesides and coworkers, which are known to affect the density and conformation of the oligoethylene glycol end‐groups on the SAM and hence their protein resistance.^[^ ^52^ ^]^ The significantly lower protein adsorption on the POEGMA brush on gold reported by Ma et al.^[^ ^10^ ^]^ is likely because the POEGMA brush provides a higher density of oligoethylene side chains compared to the most resistant ***B‐M‐E*** coating identified in this study. Despite this caveat, we believe our approach presents a valuable strategy for creating nonfouling surfaces on gold. These recombinantly produced coatings are readily adaptable to display protein‐based functionalities, enabling enzymatic activity or sensing capabilities that support diagnostic applications.

### 2.5 Adhesion of *E. Coli*

**Figure 4:**
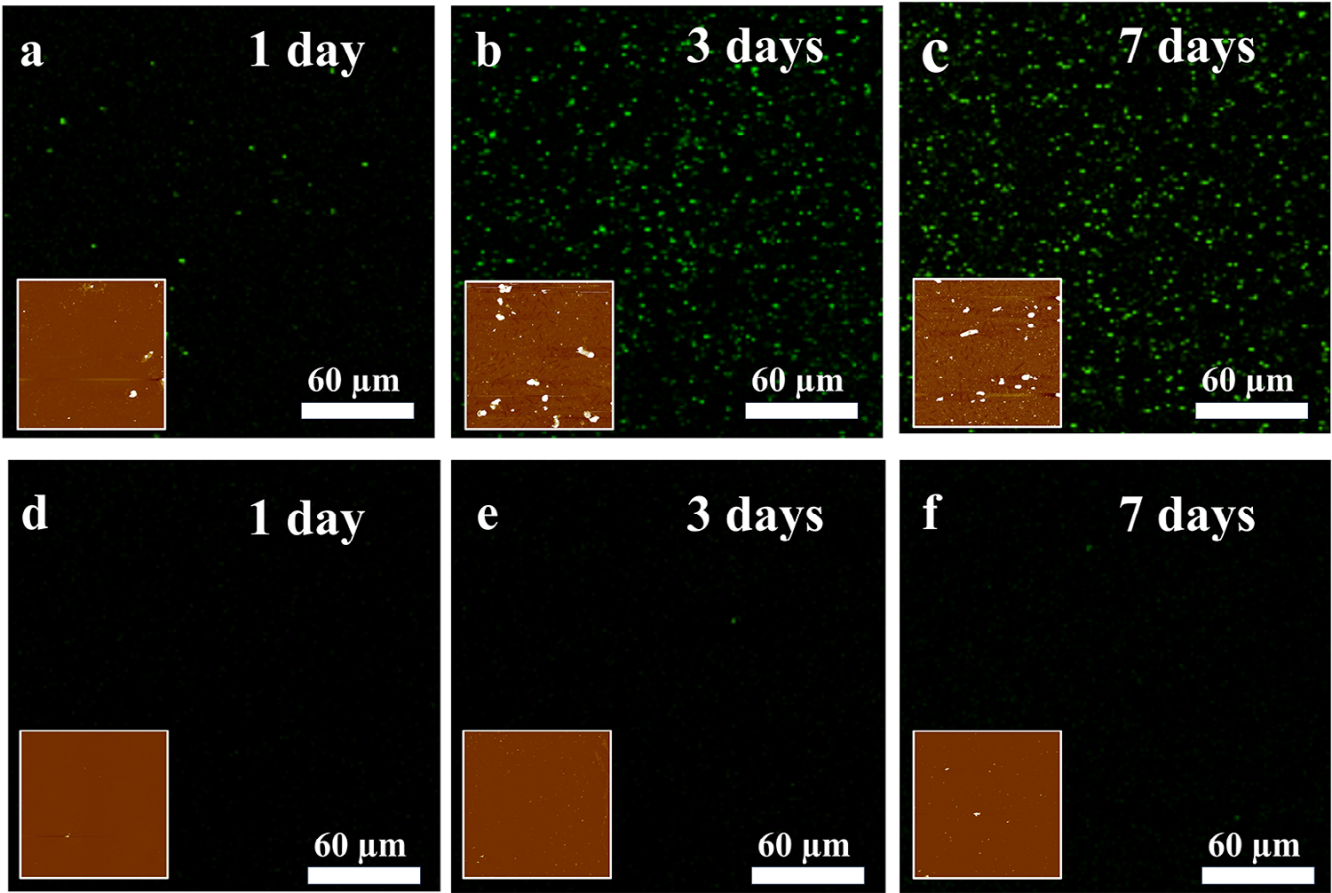
Representative CLSM images and AFM images (insets) for fouling experiments with gold surfaces and *E. coli* bacteria. a–c) Transparent gold‐coated glass slides incubated with *E. coli* in LB medium supplemented with kanamycin for a) 1 day, b) 3 days, c) 7 days. d–f) ***B‐M‐E*** *^IDP^* ^3^ coated transparent gold‐coated glass slides incubated with *E. coli* in LB medium supplemented with kanamycin for d) 1 day, e) 3 days, f) 7 days. The scale bar for CLSM images is 60 µm. AFM images are 30 µm *×* 30 µm with a height scale of 200 nm. The same samples were used for CLSM and AFM measurements.

Finally, we tested the performance of ***B‐M‐E*** *^IDP3^* coatings on gold surfaces against the adhesion of *E. coli*. For these experiments, we used *E. coli* expressing green fluorescent protein (GFP) to aid visualization of the surfaces using confocal laser scanning microscopy (CLSM). We also used atomic force microscopy (AFM) to visualize the surface fouling degree. For CSLM, we used glass slides that were coated with a thin and transparent layer of gold to enable the cells to be imaged. Bare gold and gold coated with ***B‐M‐E*** *^IDP3^* were incubated in a culture of *E. coli* transformed with a GFP expressing plasmid in Luria‐Bertani (LB) broth supplemented with kanamycin for 7 days. Samples were withdrawn from the culture on days 1, 3, and 7, extensively washed with Milli‐Q water to remove weakly attached bacteria, and then imaged by CLSM and AFM after drying. **Figure** **4**a–c shows representative CLSM images of bare gold surfaces after incubation with GFP‐expressing *E. coli* for 1 day, 3 days, and 7 days, respectively. Corresponding images for ***B‐M‐E*** *^IDP3^* coated gold surfaces are shown in Figure 4d–f. Additionally, the average fluorescence intensities are given in Figure S9a (Supporting Information). These images qualitatively show that the ***B‐M‐E*** *^IDP3^* coating reduces *E. coli* adsorption compared to bare gold.

We also imaged bare gold and ***B‐M‐E*** *^IDP3^* coated gold surfaces using AFM after drying. We first perform a reference measurement for the ***B‐M‐E*** *^IDP3^* coating on ultra‐flat gold substrates (Figure S10a,b,d,e, Supporting Information) and transparent gold substrates (Figure S10c,f, Supporting Information). Similar to what we have shown previously for other ***B‐M‐E*** proteins,^[^ ^26^, ^27^, ^28^ ^]^ the high‐resolution AFM images of ***B‐M‐E*** *^IDP3^* layers on ultra‐flat gold demonstrate that the adsorbed layer appears highly uniform, with no signs of roughness that would suggest multilayer formation or heterogeneous adsorption.

Next, at a lower spatial resolution, we imaged the ***B‐M‐E*** *^IDP3^* coated or uncoated gold surfaces incubated with *E. coli* for varying times. The same transparent gold‐coated substrates were used for AFM imaging as for the CLSM experiments. Representative AFM images are shown as insets in Figure 4. At a qualitative level, the impact of the ***B‐M‐E*** *^IDP3^* coating is clear, with many fewer bacteria adhering to the coated slides than to the bare gold slides. Figure S9b (Supporting Information) shows the quantitative analysis of the AFM images. Control AFM images of the bare gold and ***B‐M‐E*** *^IDP3^* coated gold surfaces before incubation with *E. coli* are shown in Figure S11 (Supporting Information). Additionally, we demonstrated using AFM that for areas not occupied by *E. coli* bacteria, there is significant fouling of the surface of bare gold by the components of the media, but not for the ***B‐M‐E*** *^IDP3^* coated surfaces (Figure S12, Supporting Information). By combining CLSM and AFM measurements, we conclude that the ***B‐M‐E*** *^IDP3^* coating is highly effective in preventing the adsorption of *E. coli* on gold surfaces, for at least up to 7 days.

## 3 Conclusion 

By testing SynIDPs previously optimized for other activities that also require high conformational freedom and a high solvation layer, we have identified new sequences that work well as antifouling ***E*** blocks when incorporated into the ***B‐M‐E*** family of coating proteins. The differences in antifouling performance of ***B‐M‐E*** with different ***E*** blocks are more pronounced when exposed to aggressive foulants such as undiluted HS. The IDP3 sequence is identified as providing the best antifouling activity when used as an ***E*** block in ***B‐M‐E*** proteins.

The ***E*** *^IDP3^* sequence, with the repeat motif of [(GAGAIP)_3_‐(GAGEIP)]_4_, is weakly negatively charged due to the repeating glutamic acid residues (E). Most proteins in HS, such as albumin and globulins, likewise, carry a negative charge at pH 7.4.^[^ ^53^ ^]^ We speculate that electrostatic repulsion between ***E*** *^IDP3^* polypeptides and serum proteins, along with the sequence's high conformational flexibility that creates steric hindrance, contributes to its excellent antifouling performance. Future studies will investigate their application for biomedical materials that are implanted in vivo, as well as coatings for diagnostics and biosensors, where minimization of background protein adsorption is useful for their function.

## 4 Experimental Section

### Construction of Expression Plasmids for Polypeptides

Full DNA and amino acid sequences of the (constant) ***B*** and ***M*** domains are given in Table S1 and S2 (Supporting Information), respectively. All the plasmids used in this study contain necessary features for recursive directional ligation by plasmid reconstruction (PRe‐RDL) cloning, as described by McDaniel et al.^[^ ^54^ ^]^ All protein constructs used in this study carry a His‐tag (six repeats of histidine, H_6_) for immobilized metal affinity chromatography. The DNA encoding ***B‐M***, with suitable overhangs for Gibson assembly, was purchased from Twist Bioscience. All other gene fragments, with suitable overhangs for Gibson assembly,^[^ ^55^ ^]^ were purchased from Integrated DNA Technologies (IDT). Back translation to obtain DNA sequences from amino acid sequences was performed on the IDT online platform (https://www.idtdna.com/codonopt) or by using the codon scrambler web server (https://chilkotilab.pratt.duke.edu/codon‐scrambler).

For the ***E*** *^Z6^* and ***E*** *^Z10^* domains, DNA sequences were constructed starting from a PRe‐RDL compatible plasmid previously developed for the expression of ***B‐M‐E*** *^Z20^*.^[^ ^27^ ^]^ DNA sequences for the ***E*** *^Z6^* and ***E*** *^Z10^* domains, with suitable overhangs for Gibson assembly, were inserted into a linearized version of this vector. The linearized vector was obtained by a Q5‐PCR reaction with forward primer 5‐GGTACGATTGTCTTCTAA‐3 and reverse primer 5‐TAATAATAATGATCTTCAGGATC‐3.

DNA sequences for ***B‐M***, ***B‐M‐E*** *^ZIPP^*, ***B‐M‐E*** *^IDP1^*, ***B‐M‐E*** *^ID1^*, ***B‐M‐E*** *^IDP2^*, and ***B‐M‐E*** *^IDP3^* were obtained starting from a PRe‐RDL compatible plasmid previously developed for the expression of ELP[V‐150].^[^ ^56^ ^]^ Here are the detailed steps to obtain the desired DNA sequence. First, a linearized vector was obtained by enzymatic digestion of the plasmid with XbaI and BamHI. Next, gene fragments with a suitable Gibson overhang were inserted into the linearized vector. In this way, PRe‐RDL compatible plasmids were obtained for individual blocks: the ***B***, ***M*** block, and for short repeats of the *ZIPP*, *IDP1*, *IDP2*, and *IDP3* sequences. To obtain plasmids for the ***E*** *^ZIPP^*, ***E*** *^IDP1^*, ***E*** *^IDP2^*, and ***E*** *^IDP3^* domains, PRe‐RDL was used (using the enzyme pairs AcuI/AlwNI and BseRI/AlwNI, followed by ligation) to make successive longer repeats of the *ZIPP*, *IDP1*, *IDP2*, and *IDP3* sequences until the desired total length of close to 100 amino acids was reached. Genes for the ***B‐M‐E*** constructs were obtained by PRe‐RDL cloning of plasmids encoding the ***B***, ***M***, and ***E*** domains (using the enzyme pairs AcuI/AlwNI and BseRI/AlwNI, followed by ligation). Note that these sequences all had an additional MSKGPG leader sequence^[^ ^57^, ^58^ ^]^ at the N‐terminus.

### Protein Expression and Purification

All plasmids were sequenced prior to starting the protein expression procedure. Plasmids containing the desired DNA sequences were transformed into BL21(DE3) *E. coli* (New England Biolabs). Glycerol stocks of BL21(DE3) *E. coli* harboring the desired genes supplemented with 50 µg mL^−1^ kanamycin were stored at −80 °C. The transformed *E. coli* were grown in 250 mL Erlenmeyer flasks with 25 mL of Terrific Broth (TB) containing 50 µg mL^−1^ kanamycin at 37 °C/215 rpm for at least 16 h as a starter culture. The starter culture was diluted in a 2 L Erlenmeyer flask with up to 1 L TB containing 50 µg mL^−1^ kanamycin. When the culture optical density at 600 nm (OD_600_) reached 0.6–0.8, IPTG (isopropylthio‐*β*‐galactoside) was added to the culture at a final concentration of 1 mM. The *E. coli* were then incubated for protein expression at 18 °C/215 rpm for ≈20 h before harvesting. After overnight protein expression, cultures were centrifuged at 4 °C/4000 rpm for 15 min to pellet the cells. Bacterial pellets were resuspended in 30 mL of cold lysis buffer (PBS with 30 mM imidazole). Next, resuspended cells were sonicated using a Q125 Sonicator (Qsonica) with a 2 s on/40 s off duty cycle at 85% amplitude. After sonication, the bacterial lysate was centrifuged at 4 °C at 13000 rpm for 30 and the supernatant was retained. Overexpressed proteins were isolated from the supernatant by IMAC on gravity columns (HisPur Ni‐NTA Resin, Thermo Scientific, for the resin, and Pierce Disposable Columns, Thermo Scientific). Before elution, the column was washed with 10 column volumes of lysis buffer. Elution was done using 1 column volume of elution buffer (PBS with 300 mM imidazole). A final polishing step was performed using size exclusion chromatography (SEC). 1 mL samples from IMAC purification were filtered using a 0.22 µm filter (Millex‐GV, Sigma) and then injected into a Superdex 200 Increase 10/300GL column (GE Healthcare). SEC was performed with a flow rate of 0.75 mL min^−1^ and 4 °C in PBS buffer pH 7.4. Protein fractions were tracked through the absorbance at 220, 230, or 280 nm. The purity of the proteins throughout the purification process was monitored using sodium dodecyl sulfate polyacrylamide gel electrophoresis (SDS‐PAGE).

### Matrix‐Assisted Laser Desorption Ionization Time‐of‐Flight (MALDI‐TOF) Mass Spectrometry (MS)

The molecular mass of the purified proteins was measured by MALDI‐TOF MS on a Bruker Autoflex LRF MALDI‐TOF MS instrument. All samples were analyzed at a concentration of 1 mg mL^−1^ in PBS. 2 µL of the sample was mixed with 2 µL of a saturated solution of sinapinic acid (SA) matrix, from which 1 µL was deposited on a ground steel MALDI plate. At least 2 different expression batches were analyzed for each protein. Aldolase was used as a reference. The data were processed using the Bruker FlexAnalysis 3.4 software.

### Circular Dichroism Analysis

Circular dichroism (CD) spectra were measured on an Aviv Model 202 instrument using 1 mm quartz sample cells (Hellma). Purified proteins were diluted to a final concentration of 10 µM in 10 mM PBS, pH 7.4. The CD spectra were obtained at room temperature from 260 to 190 nm in 1 nm steps with a 2 s averaging time. The CD spectra were corrected for the 10 mM PBS buffer signal at room temperature, and their average was calculated based on 3 runs.

### Quartz Crystal Microbalance with Dissipation (QCM—D) Monitoring

Gold‐coated quartz sensors (QS‐QSX301) were purchased from Biolin Scientific, Sweden. QCM data were obtained using a Q‐Sense E4 QCM‐D instrument (Biolin Scientific, Sweden). ***B‐M‐E*** protein solutions were diluted to 10 µM in PBS buffer and filtered with a 0.22 µm pore size filter. All QCM‐D measurements were performed at a flow rate of 50 µL min^−1^. First, a stable QCM‐D baseline was obtained by prolonged flushing of the QCM‐D channels. This was done until the frequency variation was less than 2 Hz. Next, ***B‐M‐E*** protein was flowed over the gold sensor for 30 min, followed by a wash with PBS for 15 min. Finally, 10% human serum (HS) for 30 min or 100% HS for 60 min was injected, followed by a wash with PBS for 15 min. The QCM‐D data were analyzed using the QSense Dfind software (version 1.2.7, Biolin Scientific, Sweden).

The calculation of the QCM‐D fouling percentage is shown in Equation (1).

(1) $$Fouling\;\;\left(\%\right)=\frac{\Delta f\;protein\;coating}{\Delta f\;bare\;gold}\times 100\%$$

where ∆*f* is the frequency shift (Hz) between two points: point 1, after applying the coating and washing with PBS but before the foulants injection; and point 2, after washing the surface challenged by foulants with PBS. Figure S5a (Supporting Information) shows the schematic diagram of the calculation of the fouling percentage.

### Atomic Force Microscope (AFM)

Transparent gold substrates (AU.0100.GC10) and ultra‐flat gold substrates (AU.1000.SWTSG) were purchased from Platypus Technologies LLC. Gold substrates were first plasma‐cleaned for 30 s and then extensively rinsed with Milli‐Q water and dried with nitrogen gas. To prepare samples for AFM imaging, 100 µL of 10 µM ***B‐M‐E*** protein in PBS was incubated on the gold surface for 30 min at room temperature. Then, samples were imaged using a Multimode AFM (Bruker, California) with the ScanAsyst imaging mode in air using Scanasyst Air cantilevers (Bruker, California) with the following specification: thickness 650 nm, length 115 µm, width 25 µm, resonance frequency 70 kHz, spring constant 0.4 N m^−1^. Data was analyzed by NanoScope Analysis version 1.5 (Bruker, California). AFM image intensity was analyzed by ImageJ.

### Dynamic Light Scattering (DLS)

A Zetasizer Nano (Malvern, UK) instrument with a scattering angle of 173° was used to measure the hydrodynamic size of gold nano particles (GNPs, 753653‐25 mL, Sigma–Aldrich) before and after protein coating. The GNP stock was diluted 100 fold with PBS prior to use. Protein samples were diluted in PBS to create a dilution series of concentrations: 0.01, 0.1, 1, 10 µM. Samples were then filtered using a 0.22 µm pore size filter and placed into a sonication bath for 5 min prior to use. Diluted GNPs and protein samples were mixed at a 1:49 (v/v) ratio of GNPs:protein. DLS measurements were performed in a quartz cuvette (105.251.005‐QS, Hellma Analytics) with a light path of 3 mm at 20°C. The DLS particle size is an average value of 3 independent replicates, where each replicate sample was measured at least 10 times by DLS. DLS data were analyzed by Zetasizer software version 7.13 (Malvern, U.K.).

### Confocal Laser Scanning Microscopy (CLSM)

A Nikon C2 CLSM was used to image *E. coli* on bare gold and ***B‐M‐E*** coated transparent gold surfaces. First, 10 mL of LB medium, supplemented with 50 µg mL^−1^ kanamycin, was used to grow GFP‐overexpressing *E. coli* overnight. The number of *E. coli* cells was counted using a spectrophotometer by their optical density at 600 nm (OD_600_). The *E. coli* suspension was diluted to a final concentration of 10^6^ cells per mL in LB medium supplemented with 50 µg mL^−1^ kanamycin and 1 mM IPTG. Transparent gold substrates (AU.0100.GC10, Platypus Technologies, LLC) were coated with ***B‐M‐E*** proteins by incubation with 10 µM protein in a 2 mL petri dish for 30 min. Then, gold substrates were incubated with 10 mL LB medium containing 10^6^ cells per mL *E. coli* in 6 well plates (Corning, USA) and were incubated at 37 °C for a specified time. CLSM image intensity was analyzed by ImageJ.

### Surface Plasmon Resonance (SPR)

A Cytiva Biacore T200 SPR (GE Healthcare Bio‐Sciences AB, Sweden) was used to measure the protein adsorption on bare gold (XanTec bioanalytics GmbH, Germany), TEG‐SAM functionalized gold (SCBS TEG, XanTec bioanalytics GmbH, Germany), and ***B‐M‐E*** *^IDP^* ^3^ (10 µM) coated gold surfaces. All SPR measurements were performed at a flow rate of 50 µL min^−1^. To coat gold with ***B‐M‐E*** *^IDP^* ^3^, the protein was injected for ≈12 min until the SPR signal saturated. This was followed by a PBS rinse until a flat base line was obtained. For the TEG‐SAM functionalized gold, the SPR experiment consisted of the following steps: in step 1, a flat baseline was obtained by flushing with PBS for at least 2 min. In step 2, 100% HS was injected for 6 min, followed by a rinse with PBS for 2 min. This step was repeated twice. For bare gold and ***B‐M‐E*** *^IDP^* ^3^ coated gold surfaces, the SPR experiment consisted of the following steps: in step 1, a flat baseline was obtained by flushing with PBS for at least 2 min. In step 2, 100% HS was injected for 7 min, followed by flushing with PBS for 2.7 min. This step 2 was repeated twice. We used the conversion factor of 1 RU = 0.1 ng cm^−2^ for the conversion of the SPR sensor response in angular units into units of mass density of the surface, following instructions of the SPR manufacturer.

### Statistical Analysis

Each experiment was performed in triplicate (*n* = 3). All data are presented as mean ± standard deviation (SD). Statistical significance between groups was assessed using One‐Way Anova with TukeyHSD online platform (https://astatsa.com/OneWay_Anova_with_TukeyHSD/). A *p*‐value < 0.05 was considered significant. All statistical analyses were conducted using Excel (Microsoft, USA).

### Ethical Statement

The genetically modified *E. coli* strains used in this study were handled in accordance with institutional biosafety regulations and approved by the local biosafety committee of Wageningen University and Duke University. Human serum was obtained from commercial sources (Sigma–Aldrich) and used in compliance with the supplier's ethical and regulatory standards. No experiments involving human participants or animals were conducted in this study.

## Conflict of Interest Statement

The authors declare no conflict of interest.

## Author Contributions

C.Z. and Y.S. contributed equally to this work. C.Z. and Y.S. performed experiments, analyzed data, and wrote the manuscript. A.V., S.D., Y.Y., and D.S. performed experiments. Y.S., Z.Z., H.Z., A.C., and R.V. supervised the project. C.Z., Y.S., A.C., and R.V. planned experiments and wrote the manuscript. The manuscript was written through the contributions of all authors. All authors have given approval to the final version of the manuscript.

## Supporting information



Supporting Information

## Data Availability

The data that support the findings of this study are available from the corresponding author upon reasonable request.
