# Supplementary material for: Nonfouling Coatings from Synthetic Intrinsically Disordered Proteins
Source: Small. 2025 Jun 30;21(34):2504365. doi: 10.1002/smll.202504365 (PMC12393014; doi:10.1002/smll.202504365)
Supplement: Supplementary file 1 — Supporting Information [file SMLL-21-2504365-s001.pdf]

NANO · MICRO
small

Supporting Information

for *Small*, DOI 10.1002/smll.202504365

Nonfouling Coatings from Synthetic Intrinsically Disordered Proteins

Chuanbao Zheng, Yulia Shmidov, Anastasia K. Varanko, Sonal Deshpande, Yunqi Yang, Daniel M. Shapiro, Zhisen Zhang, Han Zuilhof, Ashutosh Chilkoti* and Renko de Vries**

Supporting information for

Nonfouling Coatings from Synthetic Intrinsically Disordered Proteins

Chuanbao Zheng,[1,2,3‡] Yulia Shmidov,[3‡] Anastasia K. Varanko,[3] Sonal Deshpande,[3] Yunqi Yang,[3] Daniel M. Shapiro,[3] Zhisen Zhang,[4] Han Zuilhof,[2,5] Ashutosh Chilkoti,[3*] and Renko de Vries[1*]*

[1]Physical Chemistry and Soft Matter, Wageningen University & Research, 6708WE, The Netherlands
[2]Laboratory of Organic Chemistry, Wageningen University & Research, 6708WE, The Netherlands
[3]Department of Biomedical Engineering, Duke University, Durham, NC 27708, USA
[4]Research Institute for Biomimetics and Soft Matter, Fujian Provincial Key Laboratory for Soft Functional Materials Research, Department of Physics, Xiamen University, 361005 China
[5]College of Biological and Chemical Engineering, Jiaxing University, Jiaxing 314001, China

E-mail:

Yulia Shmidov yulia.shmidov@duke.edu

Ashutosh Chilkoti chilkoti@duke.edu

Renko de Vries renko.devries@wur.nl

Table S1. DNA sequences of the ***B-M-E*** components.

**Component name**	**DNA sequences**
B	ATGAGCAAAGGACCAGGTCATCACCATCACCATCATTCGAGCGGAATGCACGGGAAAACACAAGCTACTTCGGGAACCATCCAGAGCGGCTCTGGCGTCCCAGGTTCCGGTGTACCTGGATCTGGCGTACCG
M	ATGATCGAGGAAGTCGTAGCTGAGATGATCGACATCCTGGCGGAGTCCAGCAAGAAAAGCATCGAGGAATTAGCGCGTGCTGCCGATAACAAGACGACCGAGAAGGCCGTGGCCGAAGCGATTGAGGAAATTGCCCGCCTGGCGACCGCAGCGATTCAGCTGATCGAGGCATTAGCCAAGAATTTGGCGTCTGAGGAATTTATGGCCCGTGCAATTTCCGCCATCGCTGAATTGGCAAAGAAGGCAATTGAAGCAATCTATCGCTTGGCTGACAATCACACCACGGACACCTTCATGGCACGCGCCATCGCGGCGATCGCGAACCTCGCCGTTACTGCAATCTTGGCTATCGCCGCATTAGCATCGAATCATACAACCGAGGAATTCATGGCGCGTGCGATTTCCGCTATTGCAGAACTCGCGAAGAAAGCCATCGAAGCCATTTATCGCCTGGCAGATAACCATACCACGGATAAATTTATGGCGGCTGCTATCGAGGCCATCGCACTGCTTGCCACGCTGGCGATTCTGGCCATTGCCCTGCTTGCGAGTAATCACACAACTGAGGAATTCATGGCCAAAGCCATTAGTGCGATTGCGGAGTTGGCGAAGAAGGCGATCGAAGCTATTTACCGTTTGGCGGATAACCACACAAGTCCAACCTATATCGAGAAGGCTATTGAGGCAATTGAGAAGATTGCGCGCAAAGCGATTAAGGCCATTGAAATGCTGGCCAAGAACATCACCACGGAAGAATACAAAGAGAAAGCAAAATCGGCGATTGATGAAATCCGGGAAAAGGCGAAGGAAGCGATCAAACGTTTAGAAGACAATCGTACC
***E**^{Z6}*	GGTGACGGAGTACCAGGGAAGGGAGTCCCAGGTGATGGAGTGCCAGGGAAGGGCGTCCCTGGCGATGGTGTTCCCGGCAAAGGAGTACCAGGC
***E**^{Z10}*	GGTGACGGAGTACCAGGGAAGGGAGTCCCAGGCGACGGAGTCCCAGGGAAAGGTGTGCCTGGAGATGGCGTCCCAGGCAAAGGAGTCCCGGGTGATGGAGTGCCAGGGAAGGGCGTCCCTGGCGATGGTGTTCCCGGCAAAGGAGTACCAGGC
***E**^{ZZ20}*	GGGGTACCTAAAGAGGGTGTGCCTAAAGACGGTGTTCCTAAGGAAGGGGTTCCAAAGGACGGAGTCCCTAAAGAAGGTGTCCCGAAAGATGGAGTACCGAAGGAGGGAGTTCCCAAGGATGGTGTACCAAAAGAAGGCGTACCCAAAGATGGGGTACCTAAAGAGGGTGTGCCTAAAGACGGTGTTCCTAAGGAAGGGGTTCCAAAGGACGGAGTCCCTAAAGAAGGTGTCCCGAAAGATGGAGTACCGAAGGAGGGAGTTCCCAAGGATGGTGTACCAAAAGAAGGCGTACCCAAAGATGG

E^{ZIPP}	GGTGTACCGAAAGAGGGCGTGCCTAAAGAAGGTGTTCCGAAG<br>GAAGGGGTGCCAAAAGAAGGCGTCCCGAAAGAGGGTGTACCG<br>AAAGAGGGCGTGCCTAAAGAAGGTGTTCCGAAGGAAGGGGTG<br>CCAAAAGAAGGCGTCCCGAAAGAGGGTGTACCGAAAGAGGGC<br>GTGCCTAAAGAAGGTGTTCCGAAGGAAGGGGTGCCAAAAGAA<br>GGCGTCCCGAAAGAGGGTGTACCGAAAGAGGGCGTGCCTAAA<br>GAAGGTGTTCCGAAGGAAGGGGTGCCAAAAGAAGGCGTCCCG<br>AAAGAGGGT
E^{IDP1}	GGACAATCGGGATTGCCTGGTCAATCAGGGCTGCCCGGCCAAT<br>CCGGTTTGCCGGGTCAGTCTGGTCTGCCTGGACAATCAGGCTTA<br>CCAGGCCAAAGTGGGCTTCCGGGGCAAAGCGGTCTGCCCGGGC<br>AGTCGGGCCTTCCCGGACAATCGGGATTGCCTGGTCAATCAGG<br>GCTGCCCGGCCAATCCGGTTTGCCGGGTCAGTCTGGTCTGCCTG<br>GACAATCAGGCTTACCAGGCCAAAGTGGGCTTCCGGGGCAAAG<br>CGGTCTGCCCGGGCAGTCGGGCCTTCCCGGG
E^{IDP2}	GGTACCCACGGCACCCCTGGTACACATGGTACGCCAGGTACCC<br>ATGGCACACCAGGCACGCATGGGACACCTGGTACCCATGGCAC<br>ACCAGGCACGCATGGGACACCTGGCACACACGGAACACCCGG<br>TACACACGGTACTCCGGGAACCCATGGCACCCCTGGTACACAT<br>GGTACGCCAGGTACCCATGGCACACCAGGCACGCATGGGACAC<br>CTGGCACACACGGAACACCCGGTACACACGGTACTCCGGGAAC<br>CCACGGCACCCCTGGTACACATGGTACGCCAGGA
E^{IDP3}	GGAGCGGGGGCGATTCCCGGTGCAGAAGCAATTCCGGGGGCA<br>GGCGCCATTCCTGGAGCGGGGGCCATCCCAGGAGCGGGGGCG<br>ATTCCCGGTGCAGAAGCAATTCCGGGGGCAGGCGCCATTCCTG<br>GAGCGGGGGCCATCCCAGGAGCGGGGGCGATTCCCGGTGCAG<br>AAGCAATTCCGGGGGCAGGCGCCATTCCTGGAGCGGGGGCCAT<br>CCCAGGAGCGGGGGCGATTCCCGGTGCAGAAGCAATTCCGGG<br>GGCAGGCGCCATTCCTGGAGCGGGGGCCATCCCAGGT

Table S2. Amino acid sequences of the ***B-M-E*** components.

**Component name**	**Amino acid sequences**
B	MSKGPGHHHHHHSSGMHGKTQATSGTIQSGSGVPGSGVPGSGVP
M	MIEEVVAEMIDILAESSKKSIEELARAADNKTTEKAVAEAIEEIARL<br>ATAAIQLIEALAKNLASEEFMARAISAIAELAKKAIEAIYRLADNH<br>TTDTFMARAIAAIANLAVTAILAIAALASNHTTEEFMARAISAIAEL<br>AKKAIEAIYRLADNHTTDKFMAAAIEAIALLATLAILAIALLASNH<br>TTEEFMAKAISAIAELAKKAIEAIYRLADNHTSPTYIEKAIEAIEKIA<br>RKAIKAIEMLAKNITTEEYKEKAKSAIDEIREKAKEAIKRLEDNRT
$\boldsymbol{E^{Z6}}$	GDGVPGKGVPGDGVPGKGVPGDGVPGKGVPG
$\boldsymbol{E^{Z10}}$	GDGVPGKGVPGDGVPGKGVPGDGVPGKGVPGDGVPGKGVPGDG<br>VPGKGVPG
$\boldsymbol{E^{ZZ20}}$	GVPKEGVPKDGVPKEGVPKDGVPKEGVPKDGVPKEGVPKDGVP<br>KEGVPKDGVPKEGVPKDGVPKEGVPKDGVPKEGVPKDGVPKEG<br>VPKDGVPKEGVPKDG
$\boldsymbol{E^{ZIPP}}$	GVPKEGVPKEGVPKEGVPKEGVPKEGVPKEGVPKEGVPKEGVPK<br>EGVPKEGVPKEGVPKEGVPKEGVPKEGVPKEGVPKEGVPKEGVP<br>KEGVPKEGVPKEG
$\boldsymbol{E^{IDP1}}$	GQSGLPGQSGLPGQSGLPGQSGLPGQSGLPGQSGLPGQSGLPGQS<br>GLPGQSGLPGQSGLPGQSGLPGQSGLPGQSGLPGQSGLPGQSGLP<br>GQSGLPG
$\boldsymbol{E^{IDP2}}$	GTHGTPGTHGTPGTHGTPGTHGTPGTHGTPGTHGTPGTHGTPGTH<br>GTPGTHGTPGTHGTPGTHGTPGTHGTPGTHGTPGTHGTPGTHGTP<br>GTHGTPG
$\boldsymbol{E^{IDP3}}$	GAGAIPGAEAIPGAGAIPGAGAIPGAGAIPGAEAIPGAGAIPGAGAI<br>PGAGAIPGAEAIPGAGAIPGAGAIPGAGAIPGAEAIPGAGAIPGAGA<br>IPG

Table S3: Calculated molecular weight and MALDI-TOF measured molecular weight.

	**Calculated Mw** (Da)	**MALDI-TOF Mw** (Da)
B-M	34488.7	34404.1
B-M-E^{Z6}	36885.3	36956.9
B-M-E^{Z10}	38613.2	38704.9
B-M-E^{ZIPP}	44700.5	44610.7
B-M-E^{IDP1}	43122.1	43076.6
B-M-E^{IDP2}	43297.8	43336.5
B-M-E^{IDP3}	42241.5	42370.4
**BSA**	66430.3	66392.5

Table S4: Calculation of ***B-M-E*** (10 μM) coating layer thickness (nm). The layer thickness h for the ***B-M-E*** coating is $h = (D - d)/2$.

**GNPs**	***B-M-E***^{ZIPP}	***B-M-E***^{IDP1}	***B-M-E***^{IDP2}	***B-M-E***^{IDP3}
d = 73.9 ± 8.3	-	-	-	-
D	104.4 ± 14.7	102.0 ± 17.5	103.4 ± 16.6	99.4 ± 12.0
h	15.3 ± 8.4	14.1 ± 9.7	14.8 ± 9.3	12.8 ± 7.3

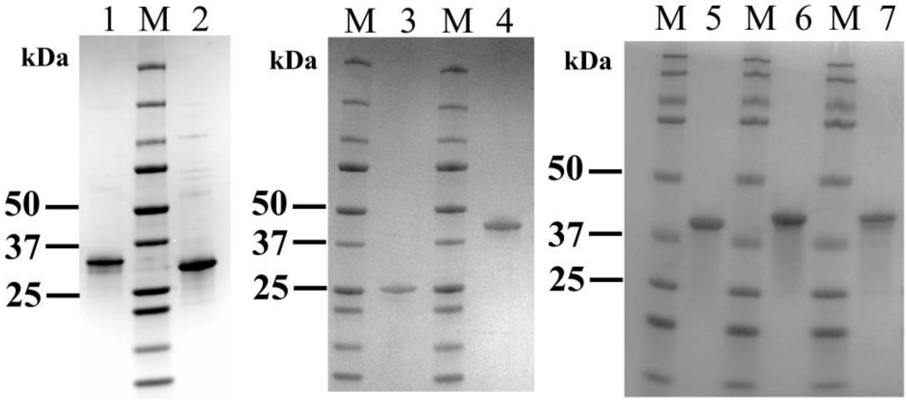

Figure S1: SDS-PAGE of purified ***B-M-E*** protein constructs. M indicates molecular marker. Lane 1: ***B-M-E***^{Z10}; lane 2: ***B-M-E***^{Z6}; lane 3: ***B-M***; lane 4: ***B-M-E***^{ZIPP}; lane 5: ***B-M-E***^{IDP1}; lane 6: ***B-M-E***^{IDP2}; lane 7: ***B-M-E***^{IDP3}. Proteins containing large stretches of intrinsically disordered regions tend to run bigger on SDS-PAGE compared to folded protein of similar size.[1,2]

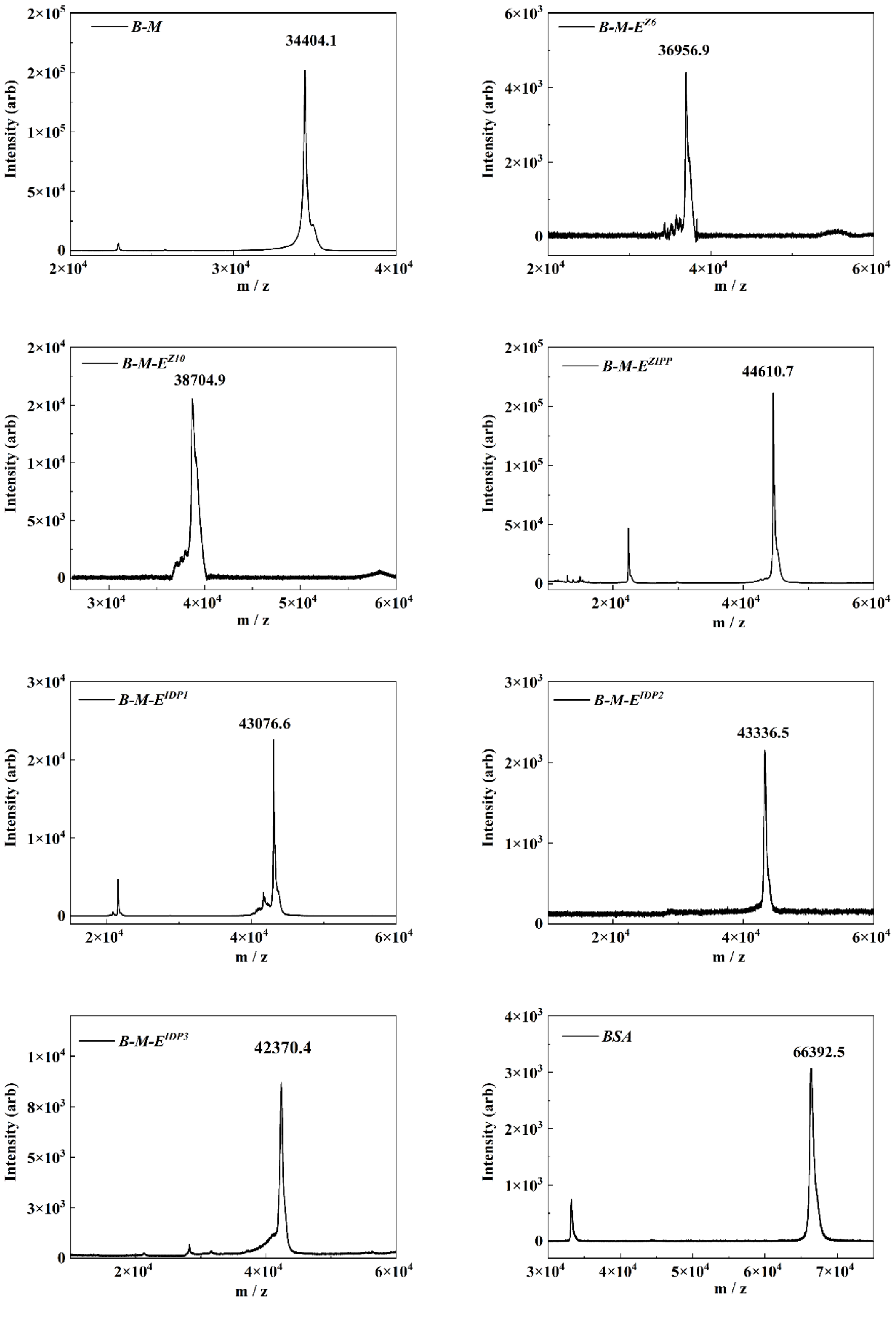

Figure S2: MALDI-TOF-MS spectra of ***B-M-E*** proteins and BSA.

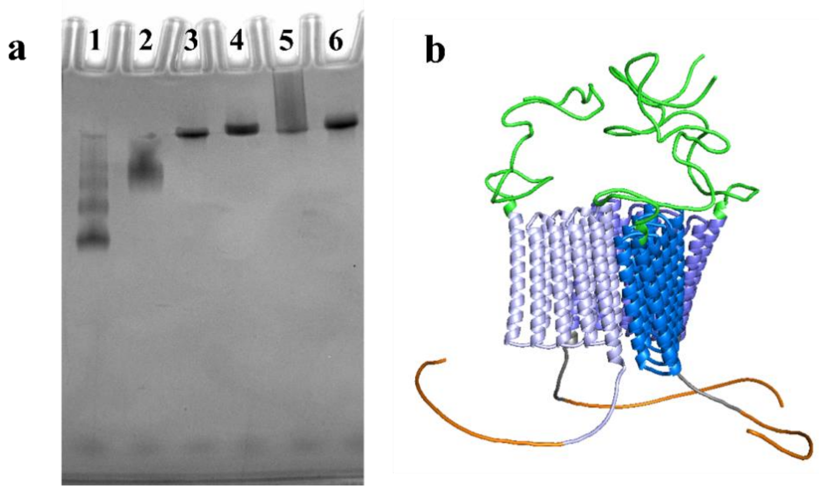

Figure S3: a) Native PAGE of BSA and purified ***B-M-E*** protein constructs. Lane 1: BSA; lane 2: ***B-M***; lane 3: ***B-M-E***^{ZIPP}; lane 4: ***B-M-E***^{IDP1}; lane 5: ***B-M-E***^{IDP2}; lane 6: ***B-M-E***^{IDP3}. The experiment was performed at pH 6.8. b) AlphaFold2 via Colab predicted ***B-M-E***^{IDP3} structure. ***B***: orange coil; ***M***: blue cartoon; ***E***: green coil. Online platform used to predict the structure: https://colab.research.google.com/github/sokrypton/ColabFold/blob/main/AlphaFold2.ipynb.[3]

Native PAGE accounts for both size and charge (as there is no SDS coating of the protein), hence a protein ladder is not applicable. Since BSA and the ***B-M-E*** constructs have similar isoelectric points, the difference in the way they run in the presence of electric field can be attributed to their size, confirming trimerization. The trimerization structure has been previously published.[4]

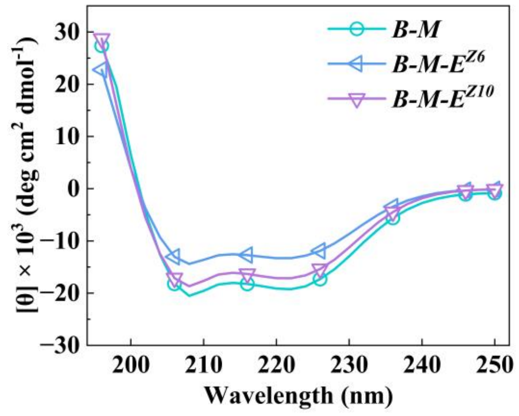

Figure S4: CD spectra of the ***B-M-E*** proteins at 10 μM in 10 mM PBS at 20 °C.

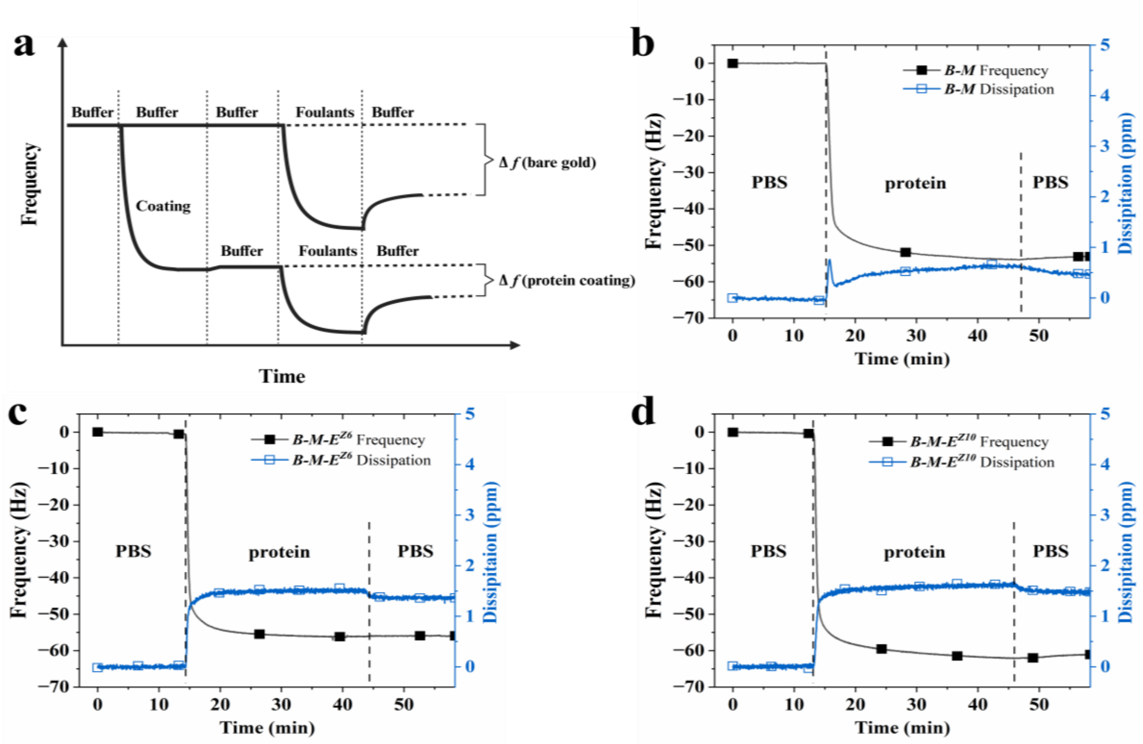

Figure S5: QCM-D frequency and dissipation data. a) Illustration of fouling percentage calculation. Coating layer formation of b) ***B-M***, c) ***B-M-E***^{Z6}, d) ***B-M-E***^{Z10} at a concentration of 10 μM in PBS.

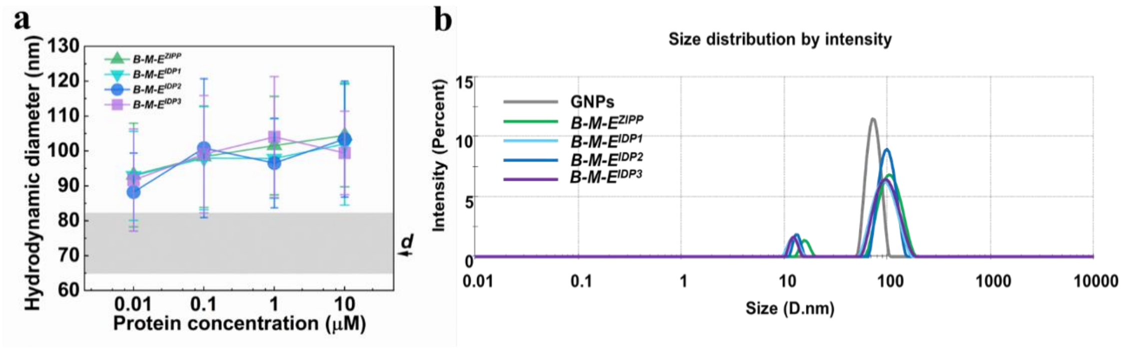

Figure S6: DLS size results by intensity for mixtures of gold nano particles and ***B-M-E*** proteins. a) Hydrodynamic diameter *D* (nm) of ***B-M-E*** coated gold nanoparticles as a function of the concentration of ***B-M-E***. The hydrodynamic diameter of the bare gold particles *d* (nm) is indicated by the gray bar (± SD). b) Size distribution by intensity of GNPs and GNPs coated with 10 μM ***B-M-E*** proteins. The peak at ~10 nm represents the excess protein in solutions.

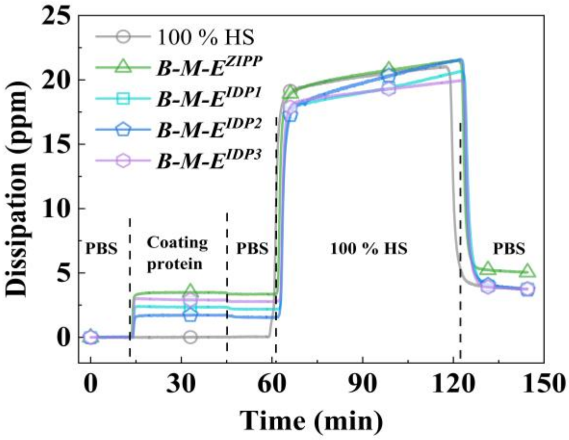

Figure S7: QCM-D dissipation data for ***B-M-E*** on gold exposed to 100% human serum.

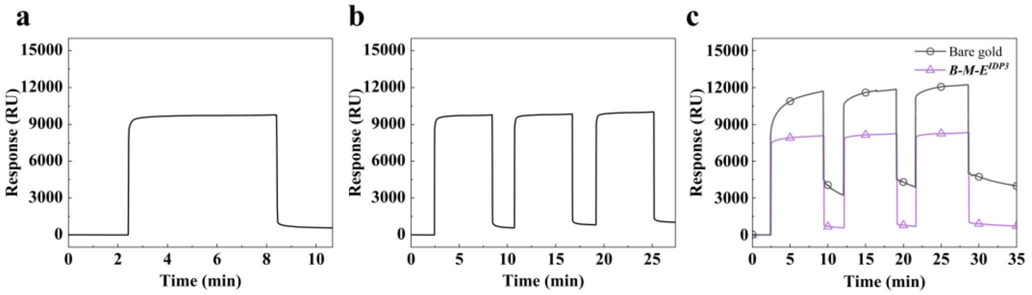

Figure S8: SPR sensorgrams of the binding of 100% human serum. a) Zoomed in sensorgrams of TEG-SAM functionalized gold surface exposed to 100% human serum.

b) Full SPR sensorgrams of TEG-SAM functionalized gold surface exposed to 100% human serum. c) Full SPR sensorgrams of bare gold (black line with circles) and ***B-M-E***^{IDP3} coated gold surface (purple line with triangles) exposed to 100% human serum.

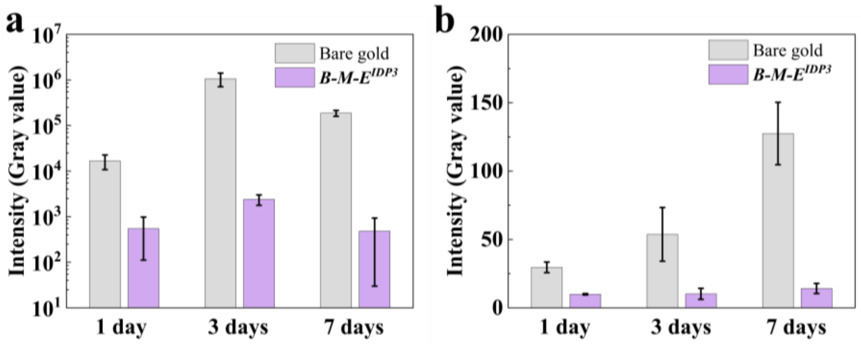

Figure S9: Intensity analysis for a) CLSM images. b) AFM images.

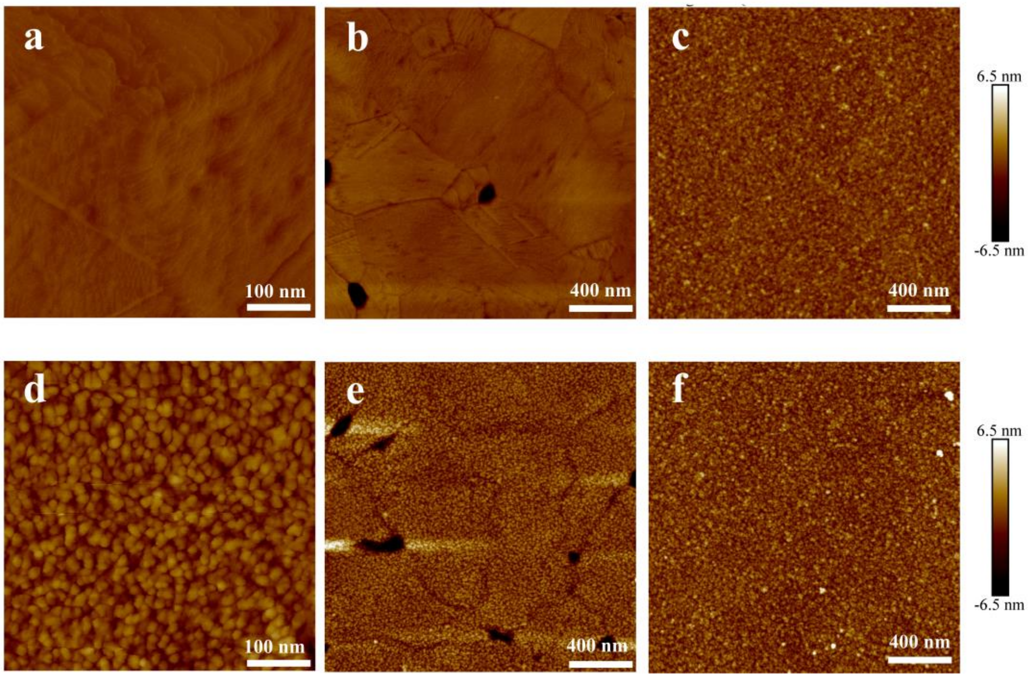

Figure S10: AFM images of ultraflat gold and transparent gold surface before and after ***B-M-E***^{IDP3} coating. a) Bare ultraflat gold. b) Zoom out image of bare ultraflat gold. c) Transparent bare gold. d) Bare ultraflat gold coated with ***B-M-E***^{IDP3}. e) Zoom out image of bare ultraflat gold coated with ***B-M-E***^{IDP3}. f) Transparent bare gold coated ***B-M-E***^{IDP3}. The scale bar is shown inside each image. All AFM measurements were performed in air.

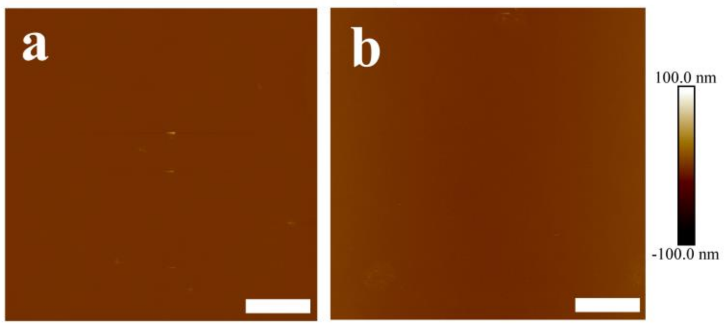

Figure S11: AFM images of a) transparent bare gold. b) transparent gold coated with ***B-M-E***^{IDP3} before mixing with LB (containing *E. coli*). The scale bar is 6 μm. All AFM measurements were performed in air.

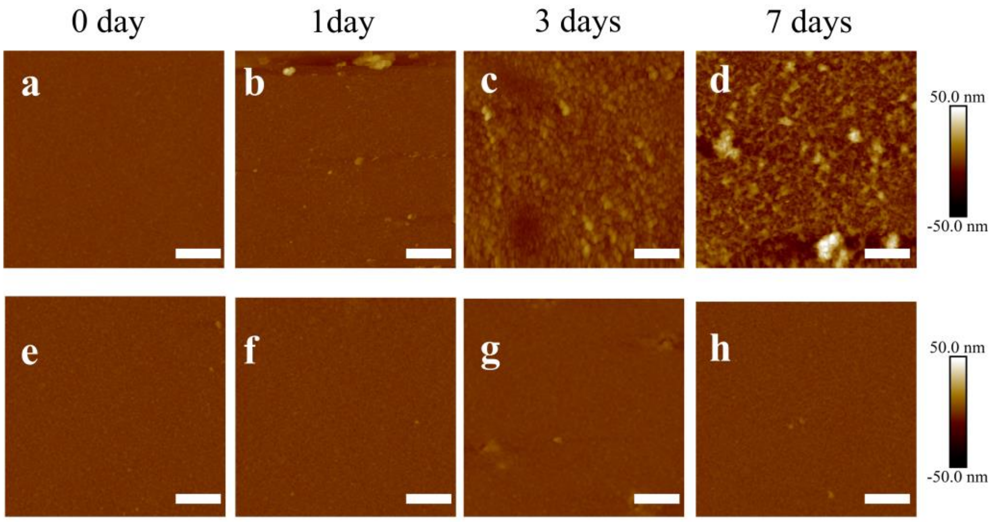

Figure S12: *E. coli* fouling experiments, AFM analysis of fouling in areas not covered by *E. coli* bacteria. AFM images for transparent bare gold a) before mixing with LB (containing *E.coli*), and after mixing with LB for b) 1 day; c) 3 days; d) 7 days, respectively. Transparent gold coated with ***B-M-E***^{IDP3} proteins e) before mixing with LB, after mixing with LB (containing *E. coli*) for f) 1 day; g) 3 days; h) 7 days, respectively. The scale bar is 400 nm. All AFM measurements were performed in air.

References

[1] S. R. MacEwan, W. Hassouneh, A. Chilkoti, *J Vis Exp* **2014**, 51583.

[2] N. C. Tang, J. C. Su, Y. Shmidov, G. Kelly, S. Deshpande, P. Sirohi, N. Peterson, A. Chilkoti, *Nat Commun* **2024**, *15*, 3727.

[3] M. Mirdita, K. Schütze, Y. Moriwaki, L. Heo, S. Ovchinnikov, M. Steinegger, *Nat Methods* **2022**, *19*, 679.

[4] J. A. Fallas, G. Ueda, W. Sheffler, V. Nguyen, D. E. McNamara, B. Sankaran, J. H. Pereira, F. Parmeggiani, T. J. Brunette, D. Cascio, T. R. Yeates, P. Zwart, D. Baker, *Nature Chem* **2017**, *9*, 353.
